# Skin pain in pediatric atopic dermatitis: mechanisms and management

**DOI:** 10.1097/ACI.0000000000001125

**Published:** 2025-12-03

**Authors:** Gabriele Simonetti, Francesca Mori, Greta Tronconi, Teresa Oranges, Romina Nassini, Lawrence F. Eichenfield, Mattia Giovannini, Francesco De Logu

**Affiliations:** a Allergy Unit, Meyer Children's Hospital IRCCS; b Department of Health Sciences, University of Florence; c Dermatology Unit, Meyer Children's Hospital IRCCS; d Department of Health Sciences, Clinical Pharmacology and Oncology Unit, University of Florence, Florence, Italy; e Departments of Dermatology and Pediatrics, University of California San Diego School of Medicine, La Jolla, California, USA

**Keywords:** atopic dermatitis, biologics, children, disease burden, pain

## Abstract

**Purpose of review:**

While itch is the hallmark symptom of atopic dermatitis (AD), skin pain is increasingly recognized as a common and burdensome symptom even in the pediatric population. Despite its prevalence, pain remains underassessed and undertreated in children with AD. This review aims to synthesize current knowledge on the prevalence, pathophysiology, clinical relevance, and management strategies of skin pain in children with AD.

**Recent findings:**

Skin pain affects up to 50% of children with AD and is associated with increased disease severity, impaired sleep, reduced quality of life, and higher rates of school absenteeism. Despite its significant impact, few studies have designed pain as a primary outcome, and validated assessment tools for young children are still lacking. Pathophysiological mechanisms involve barrier disruption, neurogenic inflammation, and cytokine-driven sensitization pathways, which may partially diverge from those implicated in itch. Emerging evidence from recent clinical trials suggests that targeted therapies can markedly reduce skin pain, sometimes independently of an improvement in visible lesions. Nonpharmacologic approaches, including emollient therapy, patient education, and psychological support, are also crucial components of a multimodal management strategy.

**Summary:**

Skin pain in pediatric AD remains an underrecognized but clinically significant symptom. Incorporating validated, age-appropriate assessment tools and integrated management strategies into clinical practice seems crucial. At the same time, further research should clarify the underlying mechanisms and refine outcome measures to guide the development of targeted therapies for affected children.

## INTRODUCTION

Atopic dermatitis (AD) is a common chronic inflammatory skin disorder characterized by xerosis, itching and eczematous lesions affecting up to 20% of children globally [1,2]. AD usually starts in childhood, and early epithelial barrier dysfunction appears to be associated with the development of other allergic and autoimmune conditions [3]. Although itch remains one of the main symptoms of AD, emerging evidence highlights the impact of skin pain on the overall burden of disease, also in the pediatric population [4,5^▪^,^6^^▪▪^]. The development of skin pain leads to sleep deprivation, irritability and impaired daytime functioning [7], affecting quality of life (QoL) for both patients and their families [8]. However, while itch is usually well evaluated in all AD patients, skin pain often remains underestimated, especially in pediatric populations, where evidence on its assessment and management remains limited.

We performed a comprehensive literature search in PubMed and Embase databases to identify articles addressing pain in pediatric AD. Our search strategy incorporated comprehensive terms for AD (including “eczema” and “atopic eczema”), pain (including “nociception”, “allodynia” and “hyperalgesia”), and pediatric populations (including “child,” “pediatric,” “paediatric,” “infant,” and “adolescen*”). Notably, when substituting pain-related terms with pruritus or itch, the databases’ search revealed a substantially higher number of publications addressing itch in pediatric AD compared to those focused on pain, highlighting a clear literature gap between the two symptoms.

This review synthesizes current evidence on the mechanism and management strategies for skin pain in pediatric AD. 

**Box 1:**
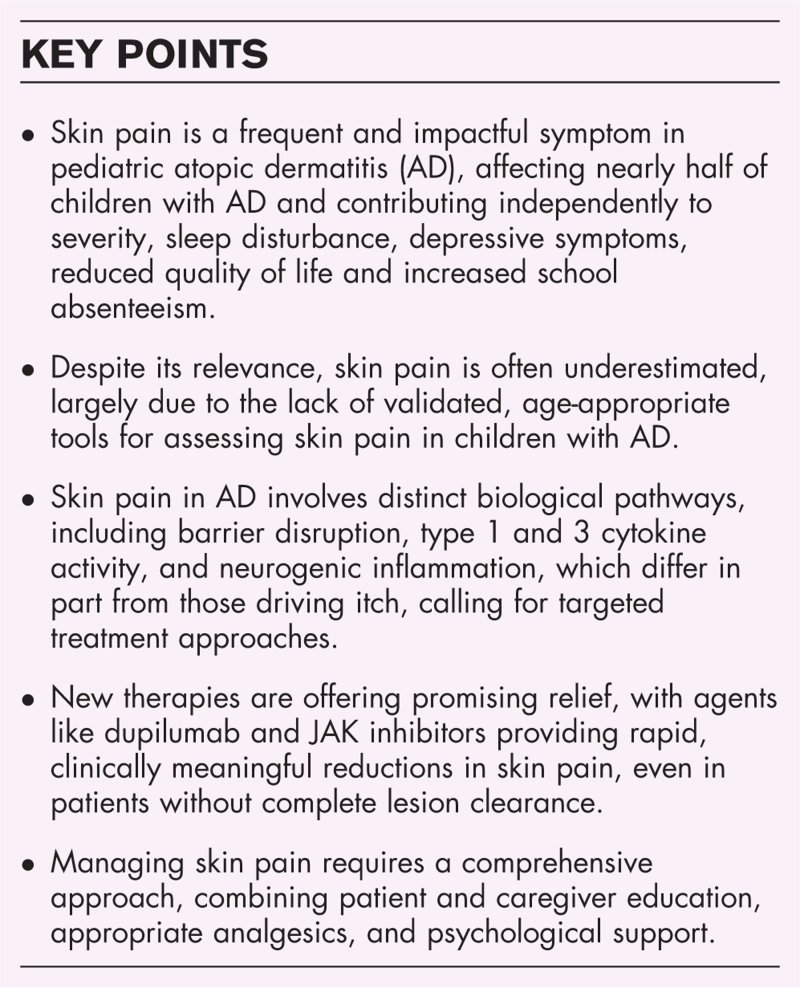
no caption available

## EPIDEMIOLOGY

AD approximately affects 15–20% of children and 1–3% of adults worldwide, with incidence rates in industrialized countries doubled or tripled over the past several decades [2,9]. Although often considered a childhood disorder, many cases persist significantly into adulthood, especially in those with early onset [10]. Clinically, AD follows a characteristic age-dependent distribution, with facial, scalp, and extensor involvement in infants and young children, and predominant flexural involvement in older children and adults [11]. There is also a variable prevalence due to geographic distribution, with over 20% of children declaring to be affected in some developed areas, while as low as 0.9% in some less developed countries such as India. In addition, low- and middle-income countries like Latin America and Southeast Asia have rising trends in AD prevalence, especially in younger children [12,13]. These trends may reflect ongoing environmental or lifestyle influences on AD development in developing economies [12].

## PAIN BURDEN IN PEDIATRIC ATOPIC DERMATITIS PATIENTS

In recent years, increasing attention has been given to the impact of skin pain in AD. While itch remains the hallmark symptom, pain is now recognized as a significant and often underestimated burden [2,4]. Inflammation, injuries from scratching, fissures, and intolerance to irritants due to AD can cause severe pain [14^▪▪^]. Moreover, atopic disorders, including AD, have been associated with nonatopic comorbidities, including chronic pain; however, their role in the development of long-term sensitization to adverse pain profiles in children remains unclear [1].

Skin pain has been extensively documented in adult patients with AD and is often associated with substantial disease burden and impaired quality of life (QoL) [15]. However, it remains frequently underestimated in pediatric patients partly due to the lack of standardized assessment tools suitable for younger children (Table 1).

**Table 1:** Summary of main clinical studies evaluating pharmacological therapies for skin pain in pediatric atopic dermatitis

Authors, year	Therapy	Population	Study overview	Pain outcome	Statistical significance
Simpson *et al.* [65], 2024	Ruxolitinib	330 children aged 2–12 years with mild-to-moderate AD	Randomized double-blind trial: • Intervention: ruxolitinib cream 0.75% or 1.5% twice daily vs. vehicle. • Duration: 8 weeks. • Assessed PROs including skin pain NRS (≥6 years).	Mean NRS skin pain score reduction at week 8: −2.9 (0.75%), −2.7 (1.5%) vs. −1.7 (vehicle)	*P* < 0.05
Simpson *et al.* [66], 2024	Upadacitinib	228 adolescents aged 12–17 years with moderate-to-severe AD	Phase III RCT (Measure Up 1 & 2): • Intervention: oral upadacitinib (15 mg or 30 mg) once daily vs. placebo. • Duration: 16 weeks. • Assessed ADerm-SS Skin Pain.	Reduction in skin pain at week 1 both for 15 mg (15.6%) and 30 mg (19.1%) vs. placebo (1.5%), sustained through week 16 (51.5–64.3% vs. 14,2%)	*P* < 0.001
Thyssen JP *et al.* [67], 2023	Abrocitinib	285 adolescents aged 12–17 years with moderate-to-severe AD	Post hoc analysis of JADE TEEN phase III RCT: • Intervention: oral abrocitinib (100 mg or 200 mg) vs. placebo • Duration: 12 weeks. • Assessed PSAAD. Skin pain.	≥4-point PSAAD reduction at week 12 both for 200 mg (51.2) and 100 mg (59.5%) vs. placebo (31.3%)	*P* < 0.05
Paller *et al.* [68], 2024	Dupilumab	162 children aged 6 months –5 years with moderate-to-severe AD	Post hoc analysis of LIBERTY AD PRESCHOOL Part B Phase III RCT: • Intervention: subcutaneous dupilumab (200 mg or 300 mg) every 4 weeks vs. placebo both with daily TCS. • Duration: 16 weeks. • Assessed caregiver-reported skin pain NRS;	Reduction of skin pain NRS significantly greater in the dupilumab group vs. the placebo group (−3.93 vs. −0.62).	*P* < 0.0001
Cork *et al.* [69], 2025	Dupilumab	41 children aged 6–11 years with moderate-to-severe AD	PELISTAD, an open-label study: • Intervention: subcutaneous dupilumab for 16 weeks based on baseline patient weight (300 mg every 4 weeks: ≥15 kg to <30 kg; 200 mg every 2 weeks: ≥30 kg to <60 kg) and matched healthy volunteers. • Duration: 16 weeks. • Assessed skin pain NRS	Reduction of skin pain NRS, which decreased from mean (SD) 6.8 (2.5) at baseline to 2.3 (1.4) at week 16	Not available

AD, atopic dermatitis; ADerm-SS, Atopic Dermatitis Symptom Scale; NRS, Numerical Rating Scale; PROs, patient reported outcomes; PSAAD, Pruritus and Symptoms Assessment for Atopic Dermatitis; RCT, randomized controlled trial; TCS, topical corticosteroids.

In a cross-sectional survey conducted by Cheng *et al.* involving 240 children with AD, skin pain was reported by 46% of parents and 44% of children aged over 8 years [5^▪^]. Skin pain intensity was assessed using a 0–10 numeric rating scale (NRS). After adjustment for disease severity, a greater pain intensity independently predicted worse QoL scores on both Infants’ Dermatitis Quality of Life (IDQOL age < 5 years) (*P* = 0.02) and Children's Dermatology Life Quality Index (CDLQI age ≥ 5 years) (*P* < 0.001).

Broader results were found by Weidinger *et al.* [6^▪▪^] in a large cross-sectional survey involving 7465 pediatric patients (aged 6  months to 18  years) with AD from 18 countries. Pain was assessed using Pain NRS (0: no pain; 10: worst possible pain). They found that patients with moderate or severe AD experienced more skin pain, frequent itch, sleep problems, impaired health-related quality of life (HRQoL) and missed more school days compared to those with mild AD. Notably, the severity of skin pain strongly correlated with overall AD severity, sleep deprivation, depressive symptoms, and poorer HRQoL.

Gorito *et al.* [16] investigated whether AD-like symptoms in early life were associated with pain at 10 years of age. The study found that children with AD-like symptoms at 6  months had a significantly higher risk for self-reporting any pain at 10 years (*P* < 0.001), and those with AD-like symptoms at 15  months had an increased risk of multisite pain reported by parents (*P* < 0.001). This study suggests that early-life AD may contribute to long-term pain sensitization, potentially through mechanisms of peripheral or central sensitization. Early management of AD could therefore play a crucial role in reducing the future pain burden in these patients.

## DIAGNOSIS AND PAIN EVALUATION IN PEDIATRIC PATIENTS

The diagnosis of AD can present challenges due to its heterogeneous clinical manifestations. Several diagnostic tools have been established to aid clinicians in identifying AD. The earliest and most widely recognized criteria were those proposed by Hanifin and Rajka [17]. More recently, the 2020 Japanese guidelines for AD aimed to simplify the diagnosis, reducing the number of criteria from over twenty to three: itch, typical morphology and distribution of lesions, and chronic or relapsing course of the disease [18]. Notably, despite all these evolving diagnostic criteria, skin pain remains excluded from major classification systems. Also, while several validated scales exist to assess pain in children [19], including the Faces Pain Scale-Revised (FPS-R) for ages 4–12 and the observational FLACC Scale (Face, Legs, Activity, Cry, Consolability) for nonverbal children, none are specific for AD pain detection. NRS evaluations of skin pain are well validated in adolescents and adults [20]; a recent study investigating the caregiver-reported skin pain NRS validated observer-reported outcomes (ObsRO) for the assessment of skin pain severity in children aged 6 months to 5 years with moderate-to-severe AD [21^▪^].

## PAIN MECHANISM IN ATOPIC DERMATITIS

Although itch remains the hallmark of AD, >40% of children experience significant skin pain [5^▪^]. These two symptoms frequently coexist [22] and share several neurobiological pathways, involving common mediators, receptors and ion channels [23].

Yosipovitch *et al.* [23] explored the overlapping and distinct peripheral mechanisms of itch and pain in AD, highlighting the high prevalence and clinical burden of both symptoms. Despite some shared pathways, distinct signaling cascades differentiate the two: itch is primarily driven by type 2 cytokines such as interleukin (IL)-4, IL-13 and IL-31 [24,25], whereas pain is more closely associated with type 1 and type 3 cytokines [e.g., IL-6, tumor necrosis factor alpha (TNF-α), IL-17A] and chemokines such as CCL2 and CXCL1 [24,26].

Skin pain in AD results from a multifactorial interplay involving skin barrier dysfunction, immune dysregulation, neurogenic inflammation, and both peripheral and central sensitization [14^▪▪^]. Disruption of the epidermal barrier facilitates the penetration of irritants and allergens, triggering abnormal sensory responses and enhanced pain perception to mild stimuli, often described as burning, stinging, or raw sensations [27]. Moreover, repetitive scratching exacerbates this condition, reinforcing a vicious cycle between itch, pain and overall discomfort [28]. Peripheral mediators, particularly those involved in inflammation, play a significant role in the development and maintenance of pain by activating peripheral neuron pathways. Lysophosphatidic acid, cathepsin S, IL-1β, IL-6, and IL-33 play a main role in sensitizing nociceptive neurons [29], while the overexpression of neurotrophic factors, including nerve growth factor (NGF), sustains a chronic pain condition [30].

### Pain peripheral sensitization

In the context of peripheral sensitization to pain, G protein-coupled receptors (GPCRs) and transient receptor potential (TRP) channels, two prominent receptor families, play a key role in mediating skin sensation [31]. During peripheral sensitization, these receptors become activated and facilitate the transmission of pain signals along nociceptive pathways. Among GPCRs, Mas-related G protein-coupled receptors (Mrgprs) are specifically expressed by primary sensory neurons and contribute to the detection of peripheral skin stimuli. In particular, MrgprD marks a distinct subset of polymodal sensory neurons that innervate the skin and are responsive to chemical, mechanical, and thermal stimuli, thus playing a role in pain sensitization [32]. Activation of MrgprD at peripheral terminals has been shown to induce dorsal root ganglion (DRG) hyperexcitability and mechanical allodynia in various experimental pain models [33]. While MrgprD-mediated pain and itch have been investigated across multiple preclinical models [34], it is unclear whether MrgprD-expressing neurons mediate pain and itch in a mutually exclusive manner, and whether the behavioral outcome is influenced more by the modality or the intensity of the stimulus.

Together with GPCRs, some members of the TRP ion channel family are broadly expressed across both sensory and nonsensory tissues, underscoring their involvement in a wide array of physiological processes [35]. TRPs function as depolarizing cation channels, primarily permeable to calcium and sodium, in nociceptive neurons, where they play a pivotal role in the detection and modulation of pain. In addition to mediating sensory transduction, TRPs are involved in neuroimmune interactions by influencing immune cell activation, cytokine release, and neurogenic inflammation. The nociceptive TRPs are typically polymodal, responding to a range of exogenous and endogenous stimuli. Physical (e.g. temperature, tension and pressure) and chemical activation make them a molecular detector of potentially harmful signals [36]. The transduction of noxious stimuli begins with the activation of these channels in the peripheral nervous system, leading to neuronal depolarization and the initiation of electrical signaling. Among different TRP members, the TRP vanilloid 1 (TRPV1), TRP ankyrin 1 (TRPA1), and TRP melastatin 8 (TRPM8) subtypes have been detected at high levels in AD lesions, suggesting their involvement in barrier dysfunction, neurogenic inflammation and associated pain [37].

TRPV1 is upregulated in cutaneous nerve fibers and keratinocytes, contributing to pain symptoms commonly experienced by patients. Activation of TRPV1 by inflammatory mediators, such as histamine, prostaglandins, and interleukins, enhances nociceptive signaling and leads to peripheral sensitization [38]. Furthermore, TRPV1 contributes to the disruption of the skin barrier and amplifies immune responses, exacerbating the inflammatory milieu characteristic of AD [39].

TRPA1 is activated by a variety of endogenous and exogenous stimuli, including reactive oxygen species, inflammatory cytokines, and a wide variety of environmental irritants [36], all of which are increased in AD. Upon activation, TRPA1 promotes the release of neuropeptides like the calcitonin gene-related peptide (CGRP) and substance P, amplifying neuroinflammation and nociceptive signaling [40]. In preclinical models of AD, TRPA1 upregulation correlates with spontaneous scratching, mechanical hypersensitivity and skin inflammation, and pharmacological inhibition of TRPA1 significantly reduces pain-related behaviors [41]. More importantly, TRPA1 distinguishes itself from other pruriceptors by its strong involvement in the transmission of pain, particularly mechanical and cold hypersensitivity, suggesting a prominent role in the neuropathic-like pain often experienced by AD patients [4].

TRPM8 is the main mediator of cold stimuli in the mammalian peripheral nervous system [42]. Beyond its function in temperature detection, TRPM8 activation also contributes to cold-induced analgesia. For instance, mild cooling of the rodent hindpaw to temperatures has been shown to reduce both mechanical and heat-induced hyperalgesia through mechanisms dependent on TRPM8 [43]. Targeting TRPM8 may therefore be a promising approach for pain relief via cooling strategies, as already suggested in AD patients [44].

Traditionally, neurons have been considered the primary cells responsible for detecting and transmitting pain signals from the peripheral to the central nervous system. However, recent research is challenging this long-standing view, revealing that nonneuronal cells also play a pivotal role in the initiation and persistence of pain. These cells express receptors such as TRP channels, enabling them to detect noxious stimuli and activate nearby neurons via paracrine signaling. This has already been demonstrated in mouse models of neuropathic, inflammatory, and cancer-related pain, where activation of TRPA1 channels expressed by Schwann cells in peripheral nerves functions as both a detector and amplifier of nociceptive signals [45,46].

Satellite glial cells surrounding DRG cell bodies become activated following nerve injury and contribute to both the onset and persistence of neuropathic pain [47]. Thus, multiple cells, including but not limited to nerve cells, could contribute to sensitization and long-term pain in AD patients.

### Pain central sensitization

Beyond peripheral mechanisms, chronic inflammation in AD also promotes central sensitization, a phenomenon where the central nervous system becomes hyper-responsive to sensory input. This process is marked by the activation of spinal cord glial cells, including microglia and astrocytes, which are stimulated through pathways such as the endothelin-1/endothelin receptor type B axis. This activation leads to an increased central production of pro-inflammatory cytokines and chemokines, amplifying nociceptive signaling and contributing to the persistence and intensification of itch and pain [48]. Interestingly, microglial activation in the spinal cord occurs earlier and is more transient than astrocytic activation. During this process, microglia release proinflammatory cytokines that sensitize dorsal horn neurons, contributing to pain [49]. Furthermore, functional neuroimaging studies have provided evidence of central involvement by revealing hyperactivation in key brain regions associated with pain perception and emotional processing, such as the anterior cingulate cortex, insular cortex, and somatosensory cortices. These findings underscore the role of central nervous system dysregulation in the pathophysiology of AD-related discomfort and suggest potential targets for therapeutic intervention beyond the skin [50].

The relationship between pain and itch is complex and bidirectional. While acute pain can transiently suppress itch [51], alterations in nociceptive circuitry, such as those observed in AD, may paradoxically amplify it [52]. Moreover, evidence suggests that the perception of these sensations evolves with age: children often interpret intense itch as a form of pain, whereas adults tend to distinguish more clearly between the two [53].

## PAIN MANAGEMENT IN PEDIATRIC ATOPIC DERMATITIS

While therapies for AD have largely focused on alleviating itch and eczema, the management of skin pain remains insufficiently explored, especially in pediatric patients. Effective management of pain in pediatric AD requires a multidisciplinary approach targeting both the inflammatory processes and the disrupted skin barrier that underpin nociceptive sensitization (Table 1).

### Patient and caregiver education

Patient and caregiver education plays a key role in reducing disease burden, including skin pain, by helping to identify, manage and avoid common triggers of AD [54^▪▪^]. Several different factors can increase disease activity, pain and itch [55]; most of them are nonallergic triggers such as sweating, chemicals and climactic factors (low humidity and cold temperature), frequent exposure to water, irritating soaps, or mechanical irritation (e.g., long nails, wool clothing) [55,56]. Even stress can worsen AD symptoms [57], while allergens and irritants, including certain foods, contact substances, and airborne particles, may further contribute to flare-ups [58].

### General pain medications

Conventional pain medications, such as paracetamol and nonsteroidal anti-inflammatory drugs (NSAIDs), are routinely used for general pain control in children, yet no studies offer stratified data on their efficacy in treating skin pain in pediatric AD. Similarly, analgesics more commonly used in adults, like opioids, gabapentin and pregabalin, are rarely used in children and must be carefully age-adjusted in terms of doses when administered in such an age range. In addition, from studies conducted on adults, it seems that patients with AD do not exhibit a higher use of common analgesic medications than their non-AD counterparts [59].

### Emollients

Emollients and barrier repair therapies form the cornerstone of baseline management in AD [60]. Although several studies have shown that emollients can decrease itch, evidence for their impact on pain remains scarce [61]. Their chief value may lie in reinforcing the skin barrier and preventing the emergence of painful lesions, rather than providing immediate analgesia upon application [61]. Regular application of emollients plays a preventive role by maintaining skin barrier function and reducing the likelihood of painful lesions, as it has been shown to decrease trans-epidermal water loss and improve skin hydration, thereby indirectly alleviating pain and itch [62,63].

### Topical corticosteroids and calcineurin inhibitors

There is no clear evidence that topical corticosteroids (TCS) or topical calcineurin inhibitors (TCI) reduce skin pain; they are mainly used to reduce itch and eczema flare-ups. TCI can sometimes cause a short-lived burning on first applications [64].

### Other topical treatments

Emerging topical agents, including the phosphodiesterase-4 (PDE-4) inhibitor crisaborole, several topical Janus kinase (JAK) inhibitors (tofacitinib, delgocitinib, ruxolitinib), and the aryl-hydrocarbon-receptor modulator tapinarof, provide antieczematous activity, with their most pronounced efficacy consistently observed in itch reduction [54^▪▪^]. Definitive evidence that these formulations alleviate skin pain is still lacking.

However, Simpson *et al*. showed that twice-daily application of ruxolitinib cream on children aged 2–11 years significantly reduced skin pain after eight weeks, with mean NRS scores decreasing by –2.9 and –2.7 points for the 0.75% and 1.5% creams, respectively, compared to –1.7 with vehicle (*P* < 0.05) [65].

### Systemic Janus kinase inhibitors

Simpson *et al.* [66], reported that once-daily upadacitinib significantly improved skin pain and other patient-reported outcomes (PROs), such as itch, sleep disturbance, emotional state, and CDLQI, in adolescents aged 12–17 years with moderate-to-severe AD, with benefits as early as week 1 and sustained results through week 16 compared to placebo (*P* < 0.001).

Similarly, Thyssen *et al.* [67] showed that abrocitinib led to a significant reduction in skin pain among adolescents aged 12–17 years with moderate-to-severe AD. Pain relief was evident from week 1 and sustained through week 12, with over half of treated patients achieving a ≥4-point reduction in Pruritus and Symptoms Assessment for Atopic Dermatitis (PSAAD) skin pain scores or reporting minimal/no pain, compared to placebo (*P* < 0.05). Notably, improvements occurred even in patients without complete skin clearance, suggesting that abrocitinib acts on pain pathways independently of visible lesion improvement.

### Dupilumab

Paller *et al.* [68] found that dupilumab, combined with low-potency TCS, provided rapid and significant reduction in caregiver-reported skin pain in children aged 6  months to 5  years with moderate-to-severe AD. Improvements were evident by week 1 and sustained through week 16, with a significantly greater reduction in pain scores compared to placebo (*P* < 0.0001). Notably, similarly to abrocitinib, skin pain reduction occurred even in children who did not achieve full skin clearance, suggesting an independent effect on pain modulation. Similar results were observed in children aged 6–11 years, with significant improvements in skin pain NRS scores after 16 weeks of dupilumab treatment [69].

### Psychosocial intervention

Central neural mechanisms and stress-related pathways are now recognized as key drivers of chronic itch, and psychosocial stress itself can exacerbate both AD severity and itch, indirectly affecting skin pain [70]. Consequently, addressing the psychological dimension has become an essential component of comprehensive AD care. Unfortunately, dermatologists and other healthcare professionals usually underestimate the psychological impact of chronic skin disorders [71]. Behavioral interventions aimed at breaking the itch-scratch cycle, including cognitive behavioral therapy (CBT) and habit reversal training, have shown promising effect [72]. Also, mindfulness meditation seems promising in patients with skin diseases in reducing pain and itch at the same time [70]. The use of psychological techniques deserves systematic evaluation, yet these approaches have not yet been integrated into everyday clinical practice.

## CONCLUSION

Skin pain in pediatric AD has recently gained increasing attention, with growing recognition of its significant impact on the overall disease burden. Nevertheless, it remains frequently underrecognized in clinical practice, with many patients reporting that it is often overlooked during routine assessments.

In addition, few studies specifically isolate pain as a primary outcome, further limiting the understanding of treatment efficacy, especially given that children often struggle to distinguish between pain and itch, sometimes using the two terms interchangeably.

Validated, age-appropriate pain assessment tools should be incorporated into both clinical trials and routine practice to capture the disease burden and treatment response fully.

**FIGURE 1:**
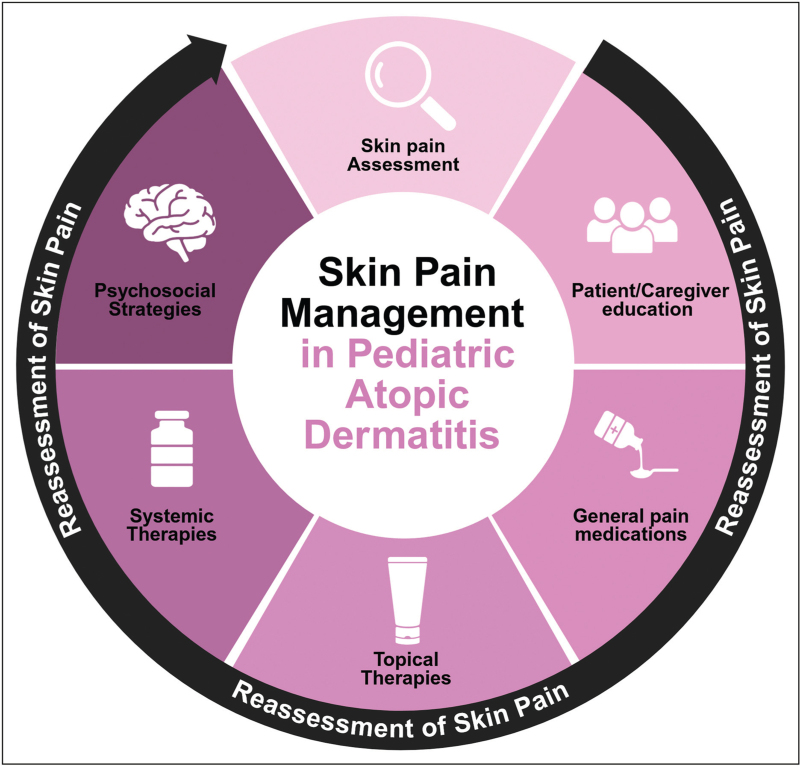
Author's proposition for skin pain management in pediatric atopic dermatitis. Created with BioRender.com.

An integrated management strategy that combines dermatologic treatments with patient education, psychological support, and targeted analgesic therapies may be more effective in addressing the multifaceted burden of pain in this population (Fig. 1).

Notably, the close relationship between pain and itch further complicates symptom evaluation and emphasized the need for standardized scoring systems. Addressing these pathways is crucial for developing effective, targeted therapies, particularly in pediatric populations where current evidence remains limited. Future research should focus on understanding the specific mechanisms and therapeutic responses to pain in AD, particularly in children.

## Acknowledgements

*The authors would like to thank Ilaria Bitossi, Alida Daniele, and Monia Marcacci from the Biomedical Library of the University of Florence for their assistance with bibliographic research.*

### Financial support and sponsorship

*None.*

### Conflicts of interest

*M.G. reports personal fees from Sanofi, Thermo Fisher Scientific. T.O. reports personal fees from Abiogen, Alexion, Novartis, Pfizer, Sanofi, Ucb. The other authors declare that they have no conflict of interests to disclose in relation to this paper.*

## REFERENCES AND RECOMMENDED READING

Papers of particular interest, published within the annual period of review, have been highlighted as:

▪ of special interest
▪▪ of outstanding interest
